# Comparison of long-term clinical outcomes of bioabsorbable polymer versus durable polymer drug-eluting stents: a systematic review and meta-analysis

**DOI:** 10.1186/s43044-024-00522-1

**Published:** 2024-07-10

**Authors:** Abdur Rehman, Ifra Eeman Ahmed, Ahmed Nouman, Rabia Irfan, Qareeha Rehman, Abdul Rehman Shah Syed, Syeda Javeria Zakir, Samar Mehdi, Maha Mushtaq Khosa, Satesh Kumar, Mahima Khatri, F. N. U. Samiullah, Tamam Mohamad, Giustino Varrassi

**Affiliations:** 1 Department of Medicine, Shaikh Zayed Hospital, Lahore, Pakistan; 2 Federal Medical and Dental College, Islamabad, Pakistan; 3 Federal Medical and Dental College, Rawalpindi, Pakistan; 4 Dow University of Health Science (Medicine), Karachi, Pakistan; 5 Quetta Institute of Medical Sciences, Quetta, Pakistan; 6 Department of Medicine, Shaheed Mohtarma Benazir Bhutto Medical College, Lyari, Karachi, Pakistan; 7 Wayne State University, Detroit, MI USA; 8 Paolo Procacci Foundation (PPF), Rome, Italy

**Keywords:** Bioabsorbable polymer BP, Durable polymer DP, Drug-eluting stent

## Abstract

**Background:**

One million individuals in the USA die from acute myocardial infarction (MI), which currently affects 3 million people globally. The available data about the early and late outcomes of both biodegradable polymer drug-eluting stents (BP-DES) and durable polymer drug-eluting stents exhibit inconsistency. We performed a meta-analysis comparing the safety and efficacy of BP-DES with DP-DES.

**Methods:**

PubMed, Google Scholar, EMBASE, Cochrane, Ovid Medline, and Clinical Trials.gov databases were used to find out studies comparing BP-DES to DP-DES. All the analyses used the random-effects model.

**Results:**

A total of 18 studies were incorporated in this meta-analysis that involved 28,874 patients, out of which 11,997 received the BP Stent, and the rest of 16,578 received the DP stent. Thorough analyses revealed that the risk of all-cause death was significantly higher in the BP-DES group (5.4% vs 2.7%) (RR 1.22, *p* 0.02) for two years or less than two-year follow-up. For studies with more than two years of follow-up, all-cause death was 9.07% (599/6603) in BP-DES and 9.47% (531/5602) in the DP-DES group but failed to achieve statistically significant levels (RR 0.97, *p* 0.58).

**Conclusions:**

The study revealed no clinically significant (*P* value was > 0.05) differences in all-cause death, cardiac death, target lesion revascularization (TLR), late stent thrombosis, device-oriented composite endpoint/target lesion failure (DOCE/TLF), myocardial infarction (MI), target vessel MI, target vessel revascularization (TVR), target vessel infarction (TVI) between BP-DES and DP-DES for more than two years of follow-up. Additionally, all-cause death was only outcomes which found to have a statistically significant difference for less than two years of follow-up, while remaining were statistically non-significant.

**Supplementary Information:**

The online version contains supplementary material available at 10.1186/s43044-024-00522-1.

## Article highlights

1. BP-DES results in a higher all-cause death rate over follow-up periods of < 2 years compared to DP-DES.
2. There are no appreciable clinical differences between the two types of stents for follow-up periods longer than 2 years for BP-DES and DP-DES.

**Supplementary Information:**

The online version contains supplementary material available at 10.1186/s43044-024-00522-1.

## Background

The prevalence of acute myocardial infarction has reached up to 3 million people around the world. Around 1 million patients die in the USA every year due to myocardial infarction (MI) [1]. MI causes permanent damage to the heart muscles and, ultimately, diastolic and systolic dysfunction in conjunction with many complications, including arrhythmias and heart failure [2]. Some of the identifiable risk factors include hypertension, dyslipidemia, smoking, and diabetes. Unfortunately, very little data are available on women and people from low ethnic backgrounds, which limits us from generalizing available data on the public [3]. Some recent reports from the World Health Organization have stated that in the next decade, most of the global burden of cardiovascular mortality will be from developing countries [3]. The emergency treatment of MI includes taking aspirin, nitroglycerin, oxygen, and some painkillers [4]. ST segment elevation MI requires restoration of blood flow, which is dealt with reperfusion therapy and angioplasty. Following a successful angioplasty treatment, nearly half of patients develop restenosis or re-occlusion within six months, and significant ischemia-related adverse events, such as repeat target vessel revascularization (TVR), can occur in up to 30% of instances [5]. It is critical to emphasize the benefits of stenting in this context, as it plays a pivotal role in reducing the occurrence of restenosis and re-occlusion, thereby minimizing the associated clinical events such as fatal re-infarction, nonfatal re-infarction, and repeat TVR in cases of recurrent ischemia [5].

The use of stents in percutaneous coronary intervention (PCI) has grown dramatically, leading to the evolution of several novel stent technologies for the treatment of symptomatic coronary disease [6]. When compared to bare metal stents (BMS), drug-eluting stents (DES) have considerably decreased the incidence of stent thrombosis, myocardial infarction, and death [7]. When first-generation stainless steel DES is compared to BMS, concerns regarding late complications such as in-stent restenosis and late stent thrombosis still exist [8]. Second-generation drug-eluting stents (DES) with enhanced stent platforms, biocompatible polymers, and more recent antiproliferative drugs were introduced to overcome the shortcomings of first-generation DES [9]. Nevertheless, neoatherosclerosis and late stent thrombosis have been connected to second-generation DES [10]. Due to prolonged re-endothelialization and delayed vascular healing, the polymer coating of drug-eluting stents has been linked to unfavorable outcomes, including stent thrombosis [11, 12]. In addition, stents that are bioresorbable or biodegradable were developed to lower the chance of restenosis.

BP-DES are safer and more effective than BMS and first-generation DES in lowering the incidence of very late stent thrombosis (ST) and restenosis [13]. Conflicting results exist on early and late stent thrombosis in DP-DES and BP-DES. Some research indicates that BP-DES carries a higher ST risk than DP-DES. Others argue that the BP-DES group has a lower ST rate than the DP-DES group [14, 15]. A recent meta-analysis of randomized controlled trials indicated no significant difference in very late stent thrombosis between two-year and five-year follow-ups for the two types of stents [16]. Verification of the safety and effectiveness of BP-DES is necessary due to the potential benefits it may provide individuals with acute myocardial infarction. This meta-analysis intends to assess the safety and efficacy of durable polymer drug-eluting stents (DP-DES) with biodegradable polymer drug-eluting stents (BP-DES), focusing on composite outcomes related to patients and devices. This study seeks to enhance the current literature by comparing BP-DES with DP-DES and presenting data to help formulate clinical recommendations for percutaneous coronary intervention with stenting.

## Methods

To conduct this meta-analysis, we followed the principles established by Preferred Reporting Items for Systemic Review and Meta-Analysis (PRISMA) [17].

### Data Source and Search Strategy

**Fig. 1:**
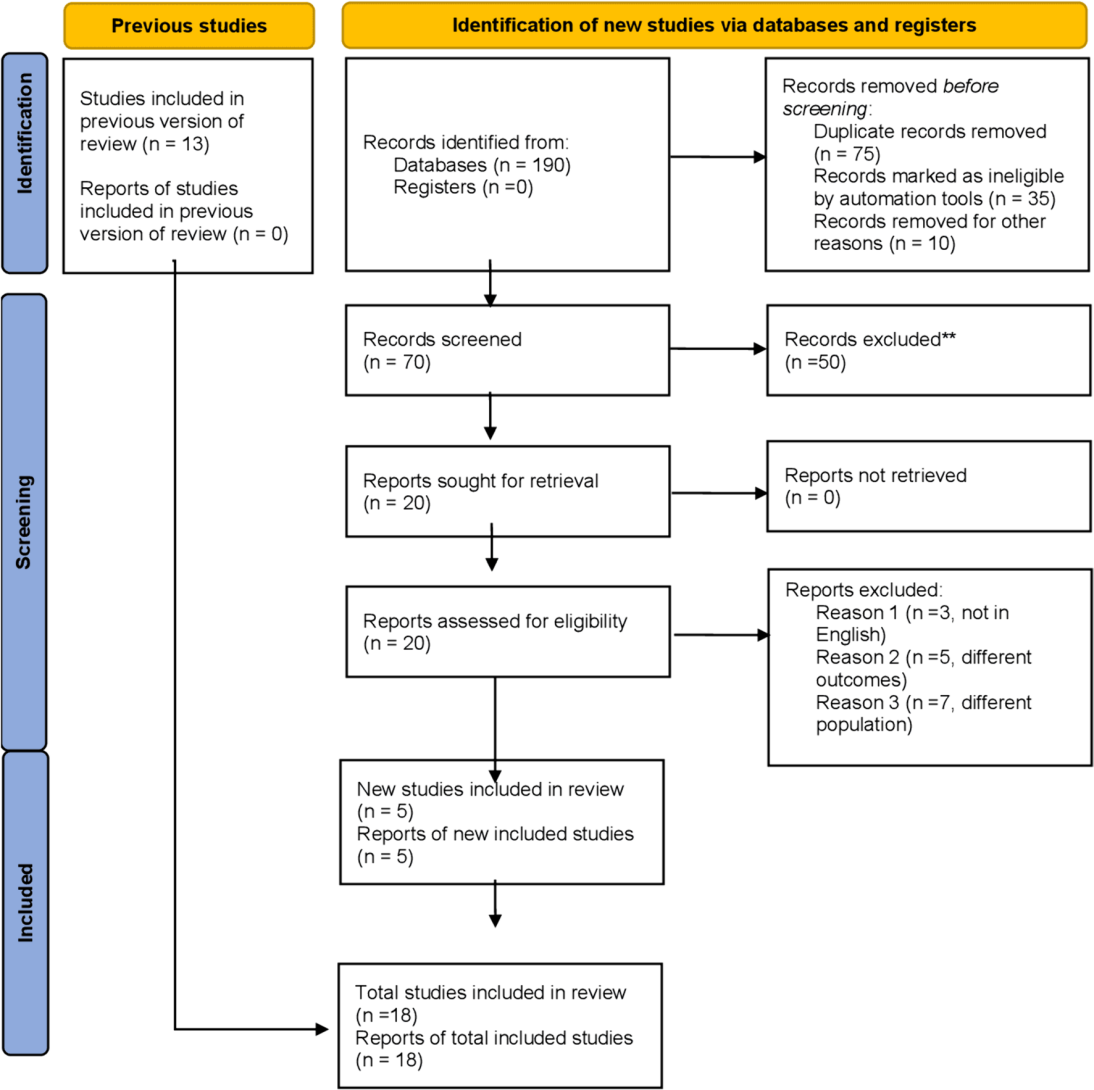
PRISMA flow chart of the included studies

An extensive search strategy was applied on the PubMed database, Google Scholar, EMBASE, Cochrane, Ovid Medline, and Clinical Trials.gov until January 4, 2023. We applied medical subject headings (MESH terms) and specific keywords for applying the search strategy [Bio absorbable polymer OR BP STENT] AND [Durable polymer or DP STENT]. The Prisma flow chart (Fig. 1) and Supplementary Table S1 contain details regarding the search strategy.

## Eligibility criteria

### Inclusion criteria

We included only those RCTs comparing outcomes between BP-DES and DP-DES. Patients who have undergone PCI were chosen, and the effect of the bioabsorbable and durable drug-eluting stent with its adverse outcomes was studied (all-cause death, cardiac death, target lesion revascularization, target vessel lesion, MI, stent thrombosis, DOCE, target vessel MI, target vessel infarction).

### Exclusion criteria

We excluded case reports, observational studies, systematic reviews, editorials, duplicates, and those studies that have different population characteristics and different outcomes. Only studies that matched our PICO were considered for considered for this analysis.

### Data extraction and quality assessment

The papers identified through the systematic search underwent exportation to the Mendeley Reference Library, where an initial screening for duplicates was conducted, and any duplicates were eliminated. Two independent reviewers, AR and SM, meticulously evaluated the remaining articles, selecting only those aligned with the pre-established criteria. The initial shortlisting of articles was based on title and abstract, followed by subsequent thorough reviews of the full papers to confirm relevance. Data extraction from the finalized reports encompassed study characteristics likes author details, publication period, study design, study participants, patients count, study rationale), clinical particulars, stent specifications, polymer type, drug details, and the drug's impact on outcomes such as all-cause death, DOCE, cardiac death, target lesion revascularization (TLR), MI, definitive, late and very late stent thrombosis, target vessel MI, target vessel revascularization (TVR), and target vessel infarction (TVI). The quality analysis of published Randomized Controlled Trials (RCTs) was conducted through the modified Cochrane’s risk of bias tool [18].

### Statistical analysis

RevMan 5.4.1, a program created in 2014 by The Cochrane Center in cooperation with The Cochrane Collaboration, was used for the statistical analysis for this meta-analysis. The analysis only included comparison studies, and the findings were displayed as weighted mean differences (WMDs) for continuous outcomes and relative risks (RRs) for dichotomous results, respectively, using forest plots. The generic inverse variance with a random-effects model was utilized to ensure accurate results. P-values were subject to a significance level of less than 0.05. For every result, a funnel plot was created to evaluate the possibility of publication bias. Heterogeneity was assessed using Higgin's I2 test, and it was classified as low, moderate, or high. Our meta-analysis had very minimal heterogeneity. All outcomes with *p*-value < 0.05 were deemed significant, and the authors carefully examined the data to ensure accuracy and dependability. The study did not seek clearance from an ethical committee because the data came from earlier clinical studies with people who gave informed consent (Tables 1, 2).

**Table 1:** Baseline characteristics of included studies

Author	Year published	Total Number	Group 1	Group 2	BP stent	Strut thickness	Drug	Polymer	Platform	DP Stent	Strut thickness	Drug	Polymer	Platform
*Studies with ≤ 2-year follow-up*														
Xu B	2011	324	168	156	TIVOLI	80	Sirolimus	PLGA	Co-Cr	Endeavor	91	Zotarolimus	Phosphoryl-choline	Co-Cr/PICr
Wijins W	2015	180	120	60	Mistent	64	Sirolimus	PLGA	Co-Cr	Endeavor	91	Zotarolimus	Phosphoryl-choline	Co-Cr/PICr
Kaiser C	2015	2256	765	765	NOBORI	120	Biolimus	PLA	Stainless steel	Xience Prime	81	Evorolimus	PVDF-HFP	Co-Cr
Jinnouchi H	2015	1132	612	520	NOBORI	120	Biolimus	PLA	Stainless steel	Xience V/PROMUS	81	Evorolimus	PVDF	Co-Cr/PICr
Jimenez VA	2016	260	126	138	ULTIMASTER	80	Sirolimus	PDLA-PCL	Co-Cr	XIENCE	81	Evorolimus	PDVF-HFP	Co-Cr
Zbiden R	2017	2048	1063	1056	Orsiro	60	Sirolimus	PLLA,Probio	Co-Cr	Xience Prime/Xpedition	81	Evorolimus	PDVF-HFP	Co-Cr
Arroyo D	2017	157	80	77	BIOMATRIX FLEX	112	Biolimus	PLLA	Stainless steel	PROMUS Element	81	Everolimus	PVDF	PI-Cr
Heijden	2018	1391	694	697	Orsino stent	60/80	Everolimus	PLGA	Co-Cr	Resolute Onyx	81	Zotarolimus	biolynx polymer system	Co-Cr
Katagiri	2020	1398	703	695	Mistent	64	Sirolimus	PLGA	Co-Cr	Xience	81	Everolimus	PVLF	Co-Cr
Baber	2021	7057	653	6404	Synergy	74	Everolimus	PLGA	Pt-Cr	Xience,Resolute	81	Everolimus,Zotarolimus	PVLF,biolynx polymer system,PVDF	Co-Cr,PLCr
Genus	2022	818	410	408	Synergy	74–81	Everlimus	PLGA	Pt = Cr	Xience	81	Everolimus	PVLF	Co-Cr
*Studies with > 2-year follow-up*														
Serruyas PW	2013	1634	857	850	BIOMATRIX FLEX	112	Biolimus A9	PLA	Stainless steel	CYPHER SELECT	140	Sirolimus	PEVA/PBMA	Stainless steel
Kufner S	2014	2374	1299	1304	YUKON CHOICE PC	87	Sirolimus	Resomer R202S	316L stainless steel	XIENCE,CYPHER	81/140	Everlimus/Sirolimus	PVDF,PEVA/PBMA	Co-Cr/PICr
Natsuki M	2015	3158	1671	1681	NOBORI	120	Biolimus A9	PLA	316L stainless steel	XIENCE,PROMUS	81	Everlimus	PVDF	CoCr/PI-Cr
Chevalier B	2015	350	238	125	NOBORI	125	Biolimus A9	PLA	316 stainless steel	TAXUS Express/Liberate	132/96	Paclitaxel	SIBS	316: Stainless steel
Meredith IT	2017	282	94	98	SYNERGY FULL +	74	Everolimus	PLGA	PI-Cr	PROMUS Element	81	Everolimus	PVDF	PI-Cr
Valchojannis	2017	2657	1795	912	NOBORI	120	Biolimus A9	PLA	316L stainless steel	XIENCE V/Prime or PROMUS	81	Everolimus	PVDF	Co-Cr/PICr
Winter	2018	1398	703	695	Mistent	64	Sirolimus	PLGA	Co-Cr	Xience	81	Everolimus	PVLF	Co-Cr

Stent information in all included studies. Natsuki et al.had a follow-up of 3 year. + Full-dose synergy stent (Boston Scientific Corp., Natick, Massachusetts). ‘Total number = total number of patients with available follow-up; ‘Group 1’ = number of patients allocated to reveive BP stents; ‘Group 2’ = number of patients allocated to receive DP stents; ‘BP’ = bioabsorbable polymer; ‘DP’ = durable polymer; ‘PLGA’ = poly(lactic-co-glycolic-acid); ‘PLA’ = polylactic acid; ‘PVDF’ = polyvinylidene

Fluoride; ‘SIBS’ = poly(styrene-b-isobutylene-b-styrene); ‘PEVA/PBMA’ = polyethylene-co-vinyl acetate(PEVA), poly-n-butylmethacrylate (PBMA); ‘Co’ = cobalt; ‘Pl’ = platinum; ‘Cr’ = chromium

**Table 2:** Meta-analysis results of all-cause death, TIF, and re-infarction comparison between BP-DES and DP-DES

BP-DES vs. DP-DES		*P* value
*Outcomes at 2 years or less than 2 years*		
All-cause death	RR 1.22, 95% CI 1.03–1.44, *I*^2^ = 0%	*P* = 0.02
Cardiac death	RR 1.11, 95% CI 0.87–1.42, *I*^2^ = 0%	*P* = 0.38
TLR	RR 1.02, 95% CI 0.88–1.19, *I*^2^ = 0%	*P* = 0.78
Late stent thrombosis	RR 0.84, 95% CI 0.59–1.2, *I*^2^ = 0%	*P* = 0.33
DOCE/TLF	RR 1.08, 95% CI 0.96–1.23, *I*^2^ = 0%	*P* = 0.20
MI	RR 1.07, 95% CI 0.83–1.39, *I*^2^ = 7%	*P* = 0.60
Target vessel MI	RR 0.98, 95% CI 0.72–1.33, *I*^2^ = 0%	*P* = 0.90
TVR	RR 1.09, 95% CI 0.77–1.54, *I*^2^ = 54%	*P* = 0.63
TVI	RR 1.03, 95% CI 0.83–1.28, *I*^2^ = 23%	*P* = 0.81
*Outcomes at more than 2 years*		
All-cause death	RR 0.97, 95% CI 0.86–1.09, *I*^2^ = 6%	*P* = 0.58
Cardiac death	RR 1.03, 95% CI 0.86–1.22, *I*^2^ = 0%	*P* = 0.75
TLR	RR 0.88, 95% CI 0.72–1.08, *I*^2^ = 56%	*P* = 0.22
Late stent thrombosis	RR 0.75, 95% CI 0.56–1.01, *I*^2^ = 0%	*P* = 0.06
DOCE/TLF	RR 1.06, 95% CI 0.92–1.21, *I*^2^ = 0%	*P* = 0.43
MI	RR 1.12, 95% CI 0.56–2.23, *I*^2^ = not applicable	*P* = 0.75
Target vessel MI	RR 1.14, 95% CI 0.55–2.38, *I*^2^ = not applicable	*P* = 0.73
TVR	RR 0.79, 95% CI 0.50–1.24, *I*^2^ = not applicable	*P* = 0.31
TVI	RR 0.99, 95% CI 0.46–2.12, *I*^2^ = not applicable	*P* = 0.98

*BP-DES* bioabsorbable polymer drug-eluting stent, *CI* confidence interval, *DP-DES* durable polymer drug-eluting stent, *M–H* Mantel–Haenszel, *RR* risk ratio, *TVR* target vessel revascularization, *TLR* target lesion revascularization

## Results

The initial product of the literature review produced a corpus of 190 articles. A subset of 18 studies [15, 19–35], all randomized controlled trials (RCTs), was found through the elimination of duplicates and rigorous evaluation of titles and abstracts. For this meta-analysis, only comparative studies were considered. A thorough search strategy was used, as shown in Fig. 1 through the PRISMA diagram. The articles in this collection span the years 2011 through 2022.

### Quality assessment and publication *bias*

The Cochrane’s Risk of Bias tool, which consists of criteria for selection, performance, detection, attrition, and reporting bias, was used to assess the quality of eligible studies. One author (S.Q.) completed the initial data collection and quality assessment; another author (X.C.) then cross-verified the procedure. Any disparities that surfaced were resolved by consulting the initial research.

### Outcomes

We evaluated a broad range of significant outcomes in our systematic review and meta-analysis comparing the long-term clinical results of BP-DES and DP-DES. These outcomes comprised of all-cause death, cardiac death, late stent thrombosis, device-oriented composite endpoint/target lesion failure (DOCE/TLF), myocardial infarction (MI), target vessel MI, TVR, and target vessel infarction (TVI). Our analysis sought a thorough understanding of the comparative performance of different stent types across these important clinical outcomes, providing valuable details about their relative efficacy and safety profiles.

#### All-cause death

**Fig. 2:**
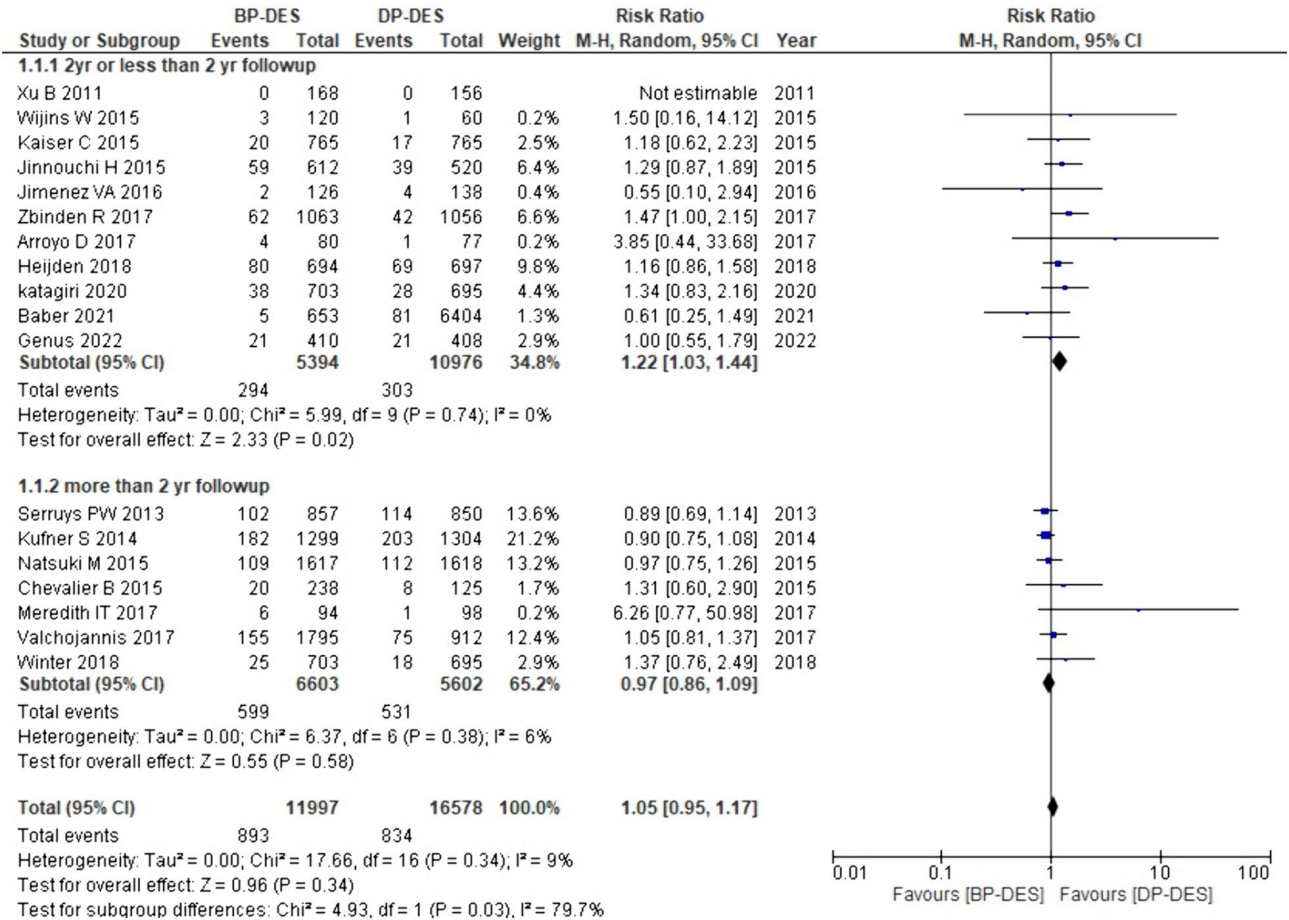
Forest plot showing all-cause death

Out of the 18 studies incorporated in our meta-analysis, data on all-cause death were provided by 17 studies. The pooled analysis showed that BP-DES is associated with an overall increased rate of all-cause death compared to DP-DES (RR: 1.05, p: 0.34). However, the findings mentioned are non-significant. Furthermore, subgroup analysis revealed that with more than two-year follow-up period, BP-DES showed a reduced risk of all-cause death in comparison with DP–DES (RR: 0.97, p: 0.58), whereas with ≤ 2-year follow-up there's a significantly higher rate of all-cause death with BP-DES (RR: 1.22, p: 0.02) (Fig. 2).

#### Cardiac death

**Fig. 3:**
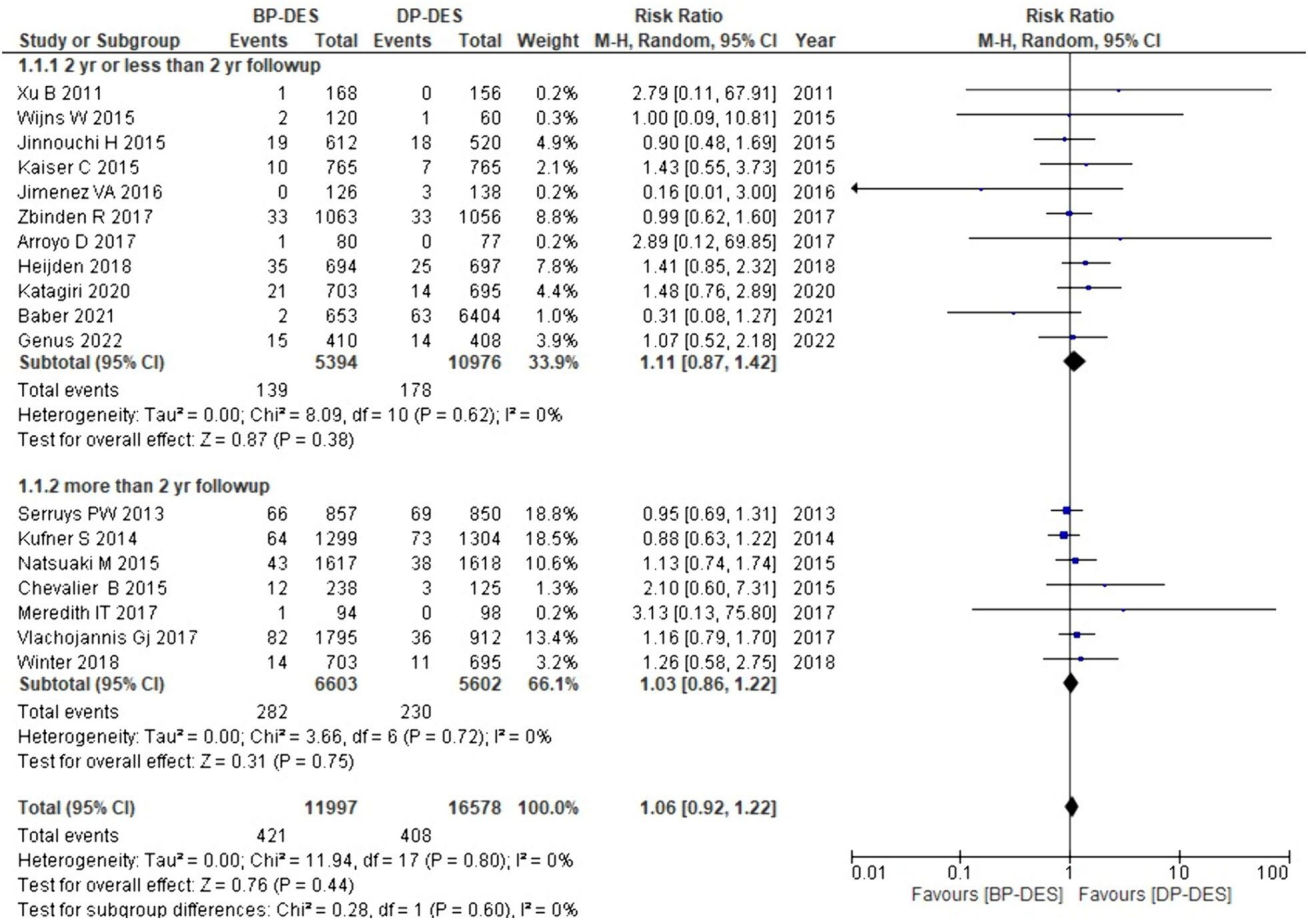
Forest plot showing cardiac death

Data on cardiac death were provided by all the included studies. The analysis revealed that compared to DP-DES, BP-DES is associated with an overall increased rate of cardiac death (RR: 1.06, *p* 0.44). Additionally, the subgroup analysis for different follow-up periods showed that with ≤ 2 years of follow-up, BP-DES is associated with an increased cardiac death rate as compared to DP-DES (RR: 1.11, p: 0.38); similarly with more than 2 year follow-up BP-DES shows the same association (RR: 1.03, p: 0.75). However, the findings mentioned above are statistically non-significant (Fig. 3).

#### Target lesion revascularization (TLR)

**Fig. 4:**
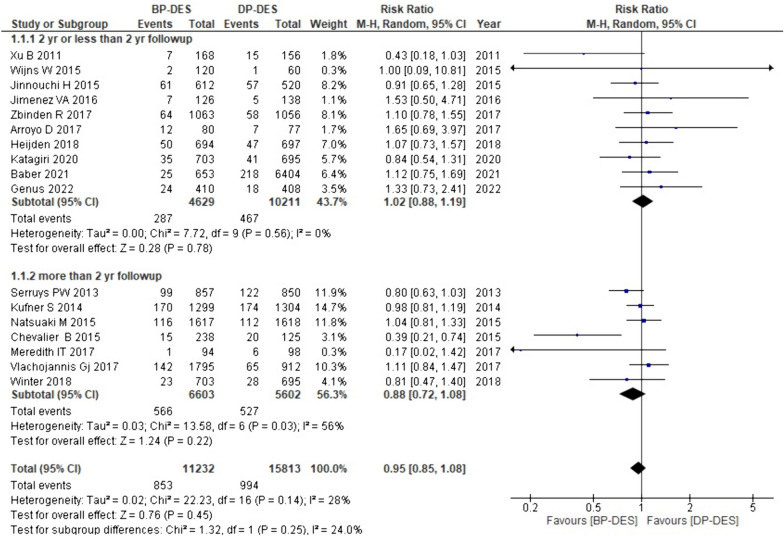
Forest plot showing target lesion revascularization

Information on TLR was acquired from all 18 studies our study. The pooled analysis generated that BP-DES has a lower overall need for TLR than DP-DES (RR: 0.95, *p* 0.45). Upon conducting subgroup analysis, with more than a 2 year follow-up period, BP-DES is associated with decreased necessity for revascularization as compared to DP-DES (RR:0.88, p: 0.22); moreover with two or more than two-year follow-up period, revascularization need is less necessary with DP-DES (RR: 1.02, *p* 0.78). However, the aforementioned findings are not statistically significant (Fig. 4).

#### Late stent thrombosis

**Fig. 5:**
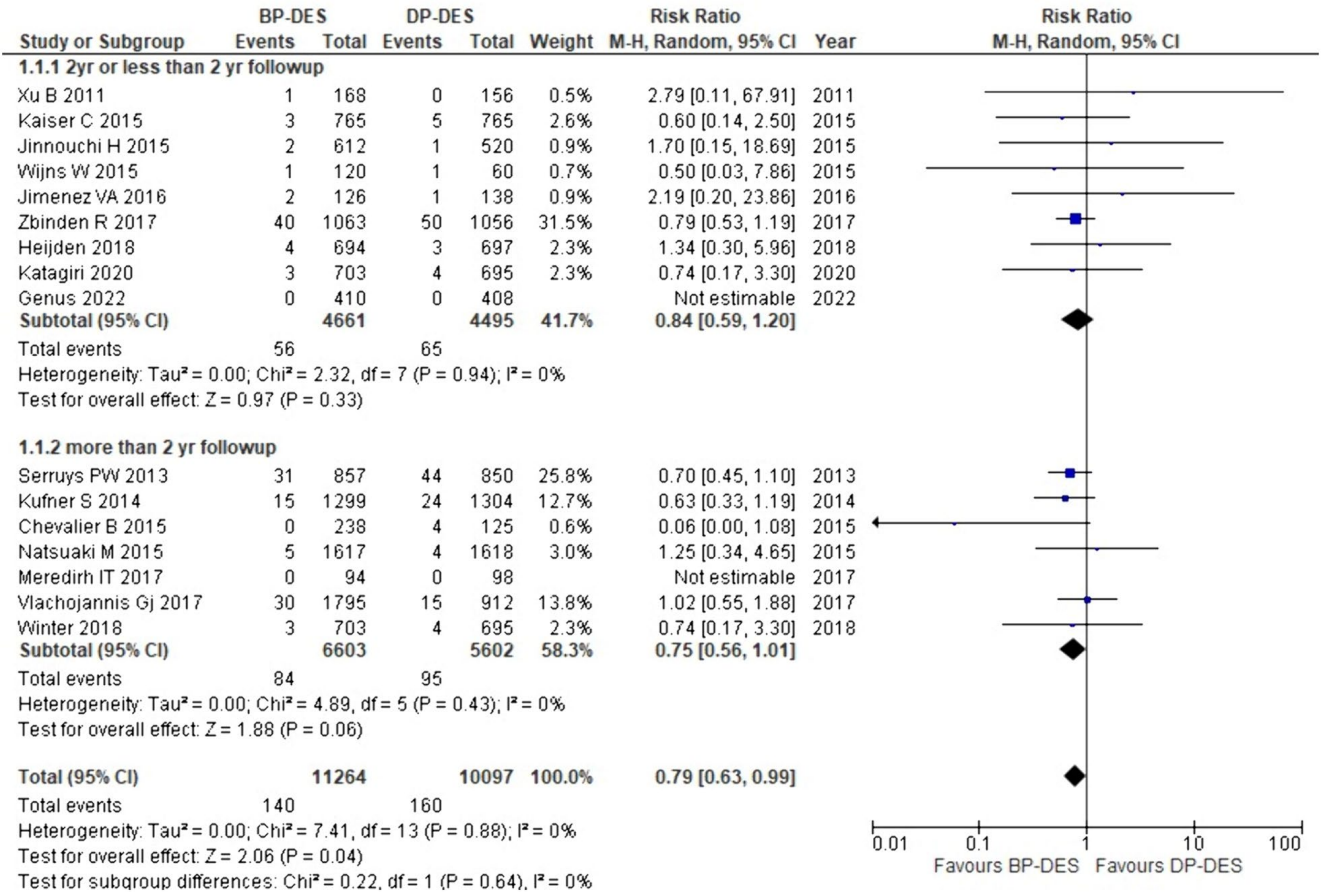
Forest plot showing late stent thrombosis

16 out of the 18 studies involved in our study provided details on late stent thrombosis. The combined analysis revealed that late stent thrombosis is significantly lowered in patients treated with BP-DES compared to DP-DES (RR: 0.79, *p* 0.04). Furthermore, it was discovered after performing a subgroup analysis that patients who receive BP-DES appear to experience late stent thrombosis less frequently than those who receive DP-DES with both two-year or less than two-year follow-up (RR:0.84, p:0.33) as well as with more than two-year follow-up (RR: 0.75, *p* 0.06) (Fig. 5).

#### Device-oriented composite endpoint/target lesion failure (DOCE/TLF)

**Fig. 6:**
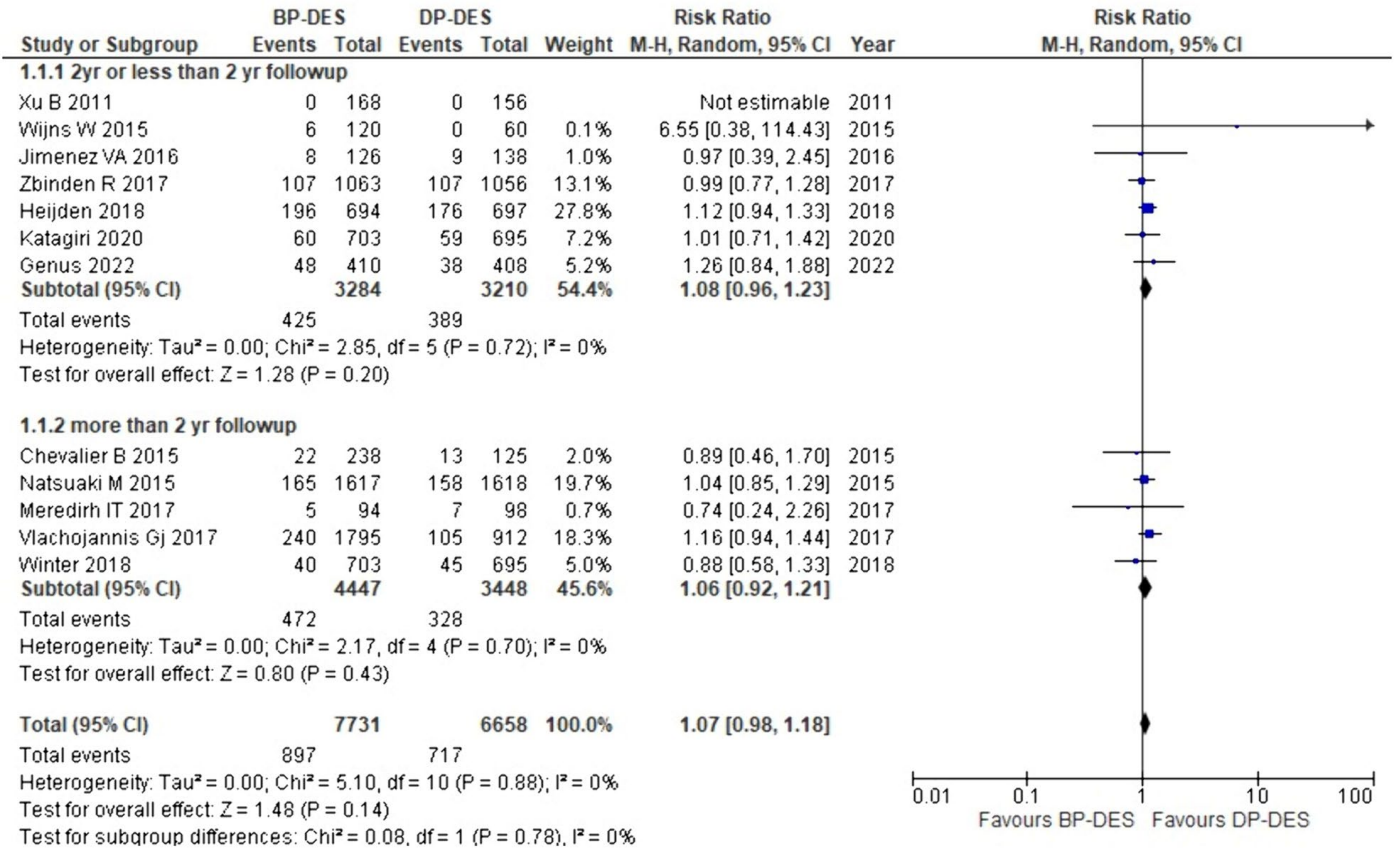
Forest plot showing device-oriented composite endpoint/target lesion failure

The rate of DOCE/TLF was reported in 11 out of 18 studies included in our study. The thorough analysis indicated a greater risk of DOCE/TLF associated with BP-DES than DP-DES (RR: 1.07, *p* 0.14). Likewise, subgroup analysis showed that with BP-DES the chances of DOCE/TLF are higher as compared to DP-DES with two years or less than two-year follow-up (RR: 1.08, p: 0.20) and with more than two-year follow-up (RR: 1.06, *p* 0.43). It is important to acknowledge that the findings presented above are insignificant (Fig. 6).

#### Myocardial infarction (MI)

**Fig. 7:**
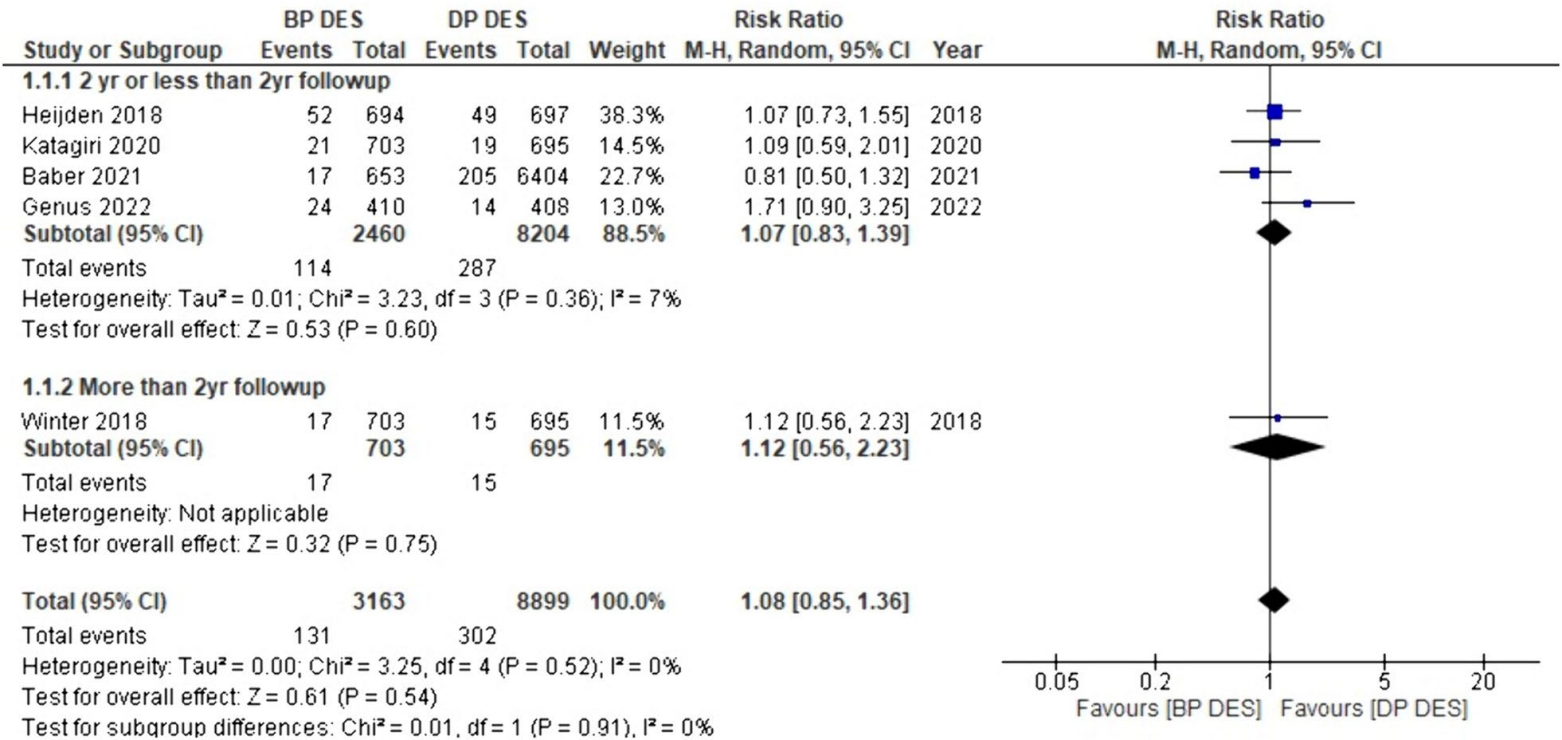
Forest plot showing Myocardial Infarction

In five of the 18 studies, data regarding myocardial infarction are provided. The combined analysis revealed a greater incidence of MI in patients receiving BP-DES compared to DP-DES (RR: 1.08, *p* 0.54). Moreover, subgroup analysis supported the same findings of higher MI occurrence with BP-DES in two years or less than two-year follow-up (RR: 1.07, *p* 0.60) and with more than two-year follow-up (RR: 1.12, *p* 0.75); however, these findings were not statistically significant (Fig. 7).

#### Target vessel MI

**Fig. 8:**
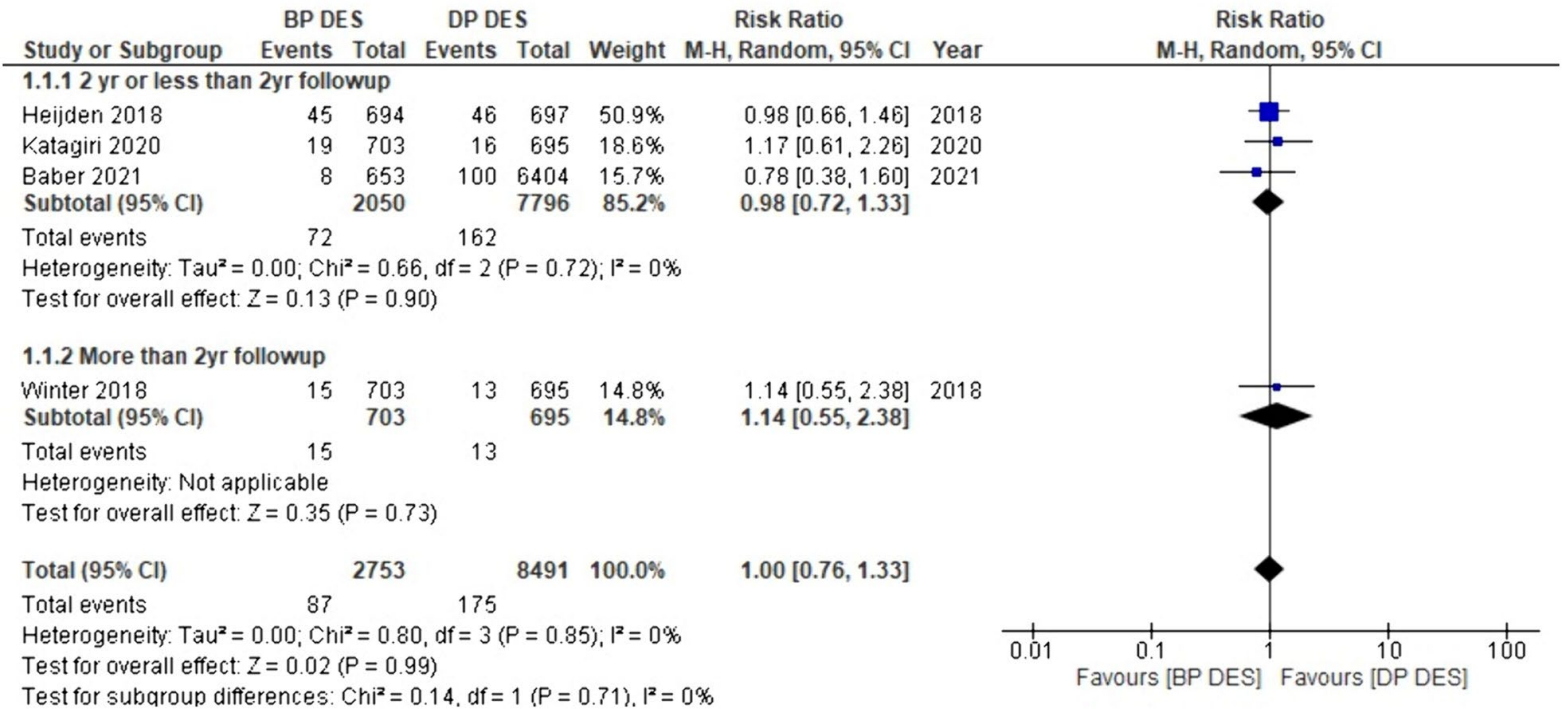
Forest plot showing target vessel MI

In this meta-analysis, data on target vessel myocardial infarction were provided by four out of the 18 studies. Interestingly, our analysis did not reveal a significant difference in the risk of target vessel MI between BP-DES and DP-DES (RR: 1.00, *p* 0.99). After conducting subgroup analysis, BP-DES was correlated with a low risk of target vessel MI with two-year or less than two-year follow-up in comparison with DP-DES (RR: 0.98, *p* 0.90), but with more than two-year follow-up risk of target vessel MI is higher with BP-DES (RR: 1.14, *p* 0.73). Nevertheless, it is essential to note that the above-mentioned findings are not statistically significant (Fig. 8).

#### Target vessel revascularization (TVR)

**Fig. 9:**
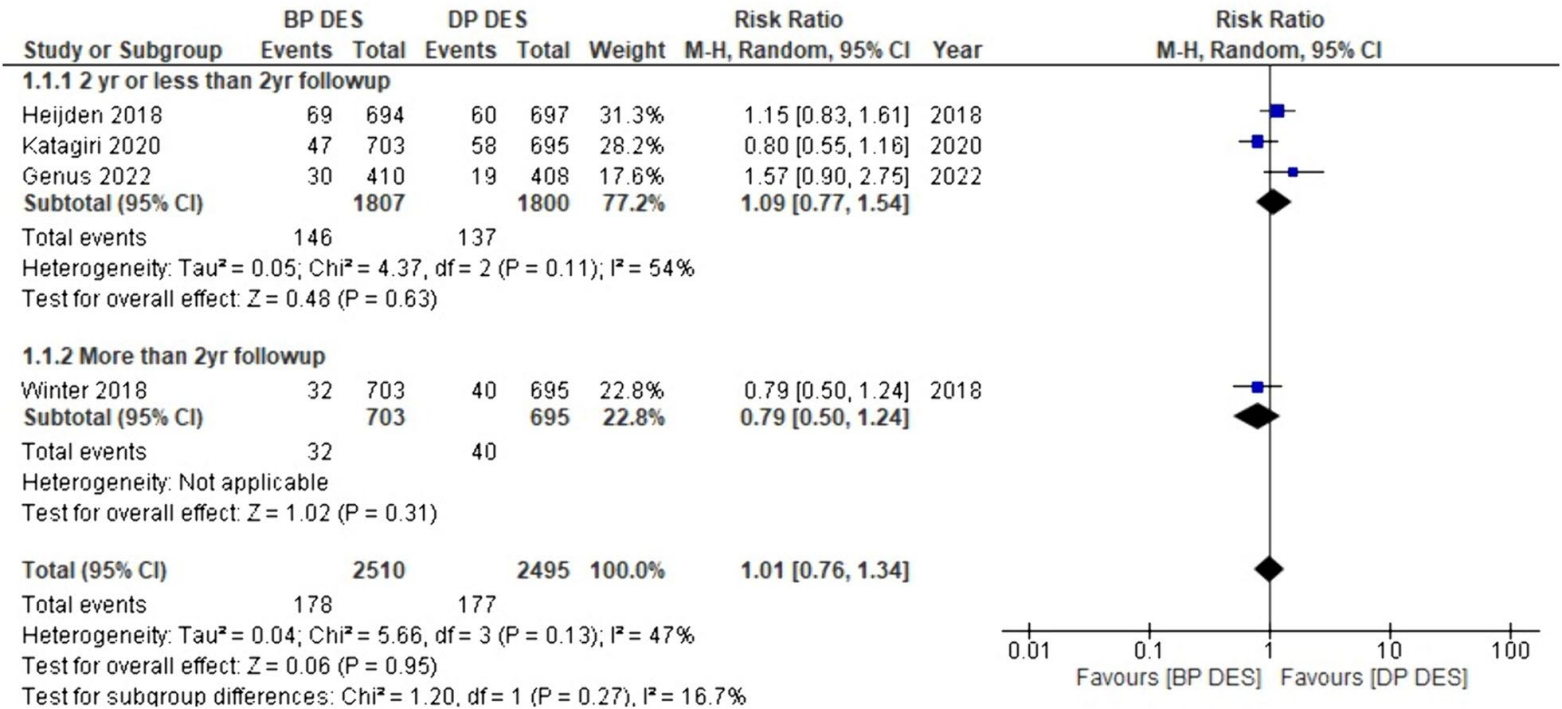
Forest plot showing target vessel revascularization

Among the 18 studies, data on TVR were available from four studies. The pooled results demonstrated that patients studied with BP-DES had slightly higher rates of target vessel revascularization than DP-DES (RR: 1.01, *p* 0.95). On top of that, subgroup analysis revealed that with more than 2-year follow-up, there is a decreased rate of TVR with BP-DES as compared to DP-DES (RR: 0.79, *p* 0.31), and with two-year or less than two-year follow-up TVR risk is higher with BP-DES (RR: 1.09, *p* 0.63). The results described above are non-significant (Fig. 9).

#### Target vessel infarction (TVI)

**Fig. 10:**
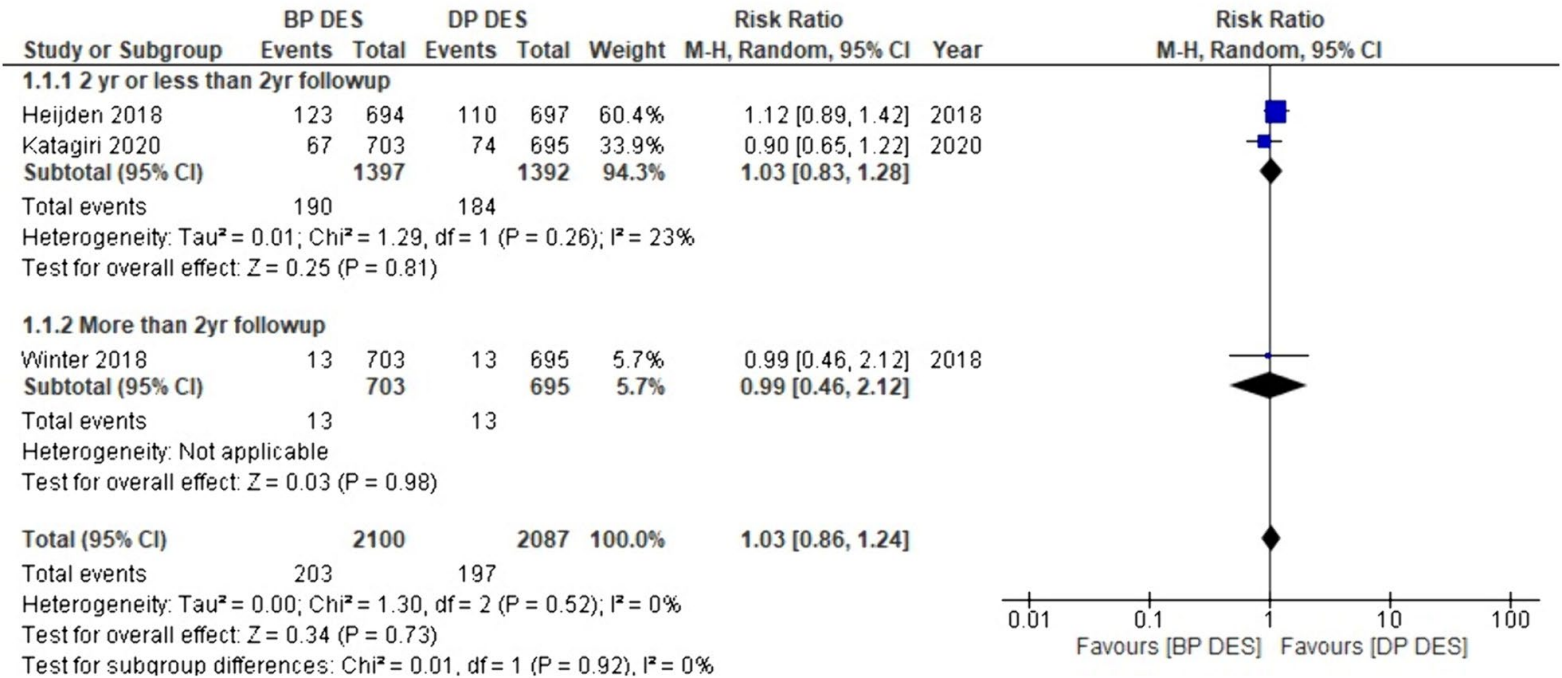
Forest plot showing target vessel infarction

Totally, 3 of the 18 studies involved our meta-analysis contributed data on target vessel infection. The combined analysis suggests a slightly greater incidence of TVI associated with BP-DES compared to DP-DES (RR: 1.03, *p* 0.73). Furthermore, sub-group analysis revealed that with two years or less than two years of follow-up, TVI incidence is higher with BP-DES (RR:1. 03, *p* 0.81); patients with follow-up exceeding two years displayed a lower risk of TVI with BP-DES (RR: 0.99, *p* 0.98) (Fig. 10).

## Discussion

Despite tremendous progress in the medical sciences, cardiovascular diseases (CVDs), such as acute myocardial infarction (AMI) and coronary artery disease (CAD), remain a major cause of mortality [1]. The advancement of drug-eluting stents (DES) in the early 2000s demonstrated a major breakthrough in the management of acute coronary syndrome (ACS). The BP-DES and the DP-DES are the two main types of these stents being compared in this topic. The platform, antiproliferative agent, and polymer coating are essential parts of DES. Lesion preparation with a balloon angioplasty catheter, guiding catheter, and coronary wire is used for stent implantation, followed by stent deployment [36]. Stent thrombosis, myocardial infarction, re-infarction, and cardiac death are among the consequences and outcomes linked to this surgery. TVI and TVR are two important concurrent outcomes. We integrated the results of five more RCTs into this evaluation. We assessed if they differed in any way from the earlier meta-analysis [16]. Based on the length of follow-up, the 18 included RCTs were divided into two sub-groups: those with follow-ups lasting two years or less and those with follow-ups lasting longer than two years.

BP-DES, which leave behind bare metal after polymer resorption, reduce complications like very late stent thrombosis and myocardial infarction [38] in contrast with DP-DES, which carry a risk of vascular inflammation [37]. Studies, however, show that compared to particular polymer durable stents, biodegradable polymer stents have a greater risk of stent thrombosis [39]. As a result, research on the relative safety and effectiveness of BP-DES and DP-DES is ongoing, requiring strong clinical backing. This meta-analysis aims to advance the clinical evidence comparing durable versus bioabsorbable DES. We have included 18 RCTs in our systematic review and meta-analysis, whose follow-up periods ranged from 12 to 60 months (5 years). Between bioabsorbable and durable DES, we could not find any significant differences in terms of device-oriented composite outcomes, such as cardiac death, target vessel MI, TVR, and target vessel infarction. Moreover, these groups had no appreciable variations in the rates of patient-oriented outcomes (revascularization, MI, and all-cause death).

During a three-year follow-up period, a recent meta-analysis found no statistically significant difference in mortality between BP-DES and DP-DES. There was no evidence of bioabsorbable DES outperforming durable DES in terms of mortality, even after a five-year follow-up [40]. Except for all-cause death, Mridha et al.'s meta-analysis revealed no differences in the clinical outcomes between BP-DESs and DP-DESs over mid- and long-term follow-ups [16]. One noteworthy finding from our meta-analysis is that it validates the superiority of DP-DES over BP-DES for all-cause death within a two-year follow-up period or less. But this dominance fades away after a follow-up period of > 2 years.

Compared to durable polymer DES, a protracted stent thrombosis—defined by the Academic Research Consortium as lasting more than a year or twelve months—has been linked to a noticeably decreased risk [41, 42]. There is conflicting information regarding extremely late ST because both types of stents showed similarities in-stent thrombosis at the two-year and five-year follow-ups, according to the most recent meta-analysis by Mridha et al. We evaluated stent thrombosis across follow-up periods of two years or longer. However, we found no statistically significant advantage of BP-DES over DP-DES. The lack of significance is probably due to variations in follow-up durations among the included RCTs. It is believed that the potential benefit of bioabsorbable polymer will become apparent following reabsorption, which might happen nine months following implantation [43]. There is no discernible difference in stent thrombosis between BP-DES and DP-DES, even with the long follow-up period of the trials in our meta-analysis.

Our meta-analysis has numerous strengths, which include all the latest RCTs up to 2022, which make the analysis more significant and up to date. This meta-analysis has a combination of all the results so that it could be easy to look for the outcomes and study them in one place. We have used diverse plots and tests, including the funnel plot, forest plot, etc. Furthermore, the result of our meta-analysis aligns with the recent meta-analysis done by Mridha et al. but contains all recently available literature. This meta-analysis supports the data that suggest the superiority of DP-DES over BP-DES in terms of all causes of death. Also, this meta-analysis shows no difference in stent thrombosis between BP-DES and DP-DES at follow-up periods of two or less than two years and even at a follow-up period of more than two years, which is consistent with the most recent meta-analysis by Mridha et al.

This analysis produced satisfactory statistical evidence, as shown in the results and figures; it is important to list some limitations. Every RCT has a different set of patient characteristics and settings. Variations in research design, intervention strategies, and baseline features of the patients, such as BMI, age, sample size, past medical history, and different struts of the stents used, may have produced some heterogeneity. Although we tried to divide the studies based on the follow-up period, still every RCT had a different follow-up time. Those with longer follow-ups can be considered more valuable. Similarly, number of participants and the ethnic group are some of the other differences among all the studies.

## Conclusions

Based on the results of this systematic review and meta-analysis, we conclude that there were no clinically significant (*P* value was > 0.05) differences between BP-DES and DP-DES for more than two years of follow-up. The only significant difference for less than two years of follow-up was all-cause death. BP-DES stent and DP-DES stent are equal in terms of long-term clinical outcomes.

### Supplementary Information

**Additional file 1**.

## Data Availability

Yes.

## Abbreviations

BP-DES: Biodegradable polymer drug-eluting stents
DP-DES: Durable polymer drug-eluting stents
MI: Myocardial infarction
TVR: Target vessel revascularization
BMS: Bare metal stent
PCI: Percutaneous coronary intervention
TLR: Target lesion revascularization
TVI: Target vessel infarction
DOCE/TLF: Device-oriented composite endpoint/target lesion failure
ACS: Acute coronary syndrome
