# Supplementary material for: Comparison of long-term clinical outcomes of bioabsorbable polymer versus durable polymer drug-eluting stents: a systematic review and meta-analysis
Source: Egypt Heart J. 2024 Jul 10;76:91. doi: 10.1186/s43044-024-00522-1 (PMC11236827; doi:10.1186/s43044-024-00522-1)

**s1. All cause death**


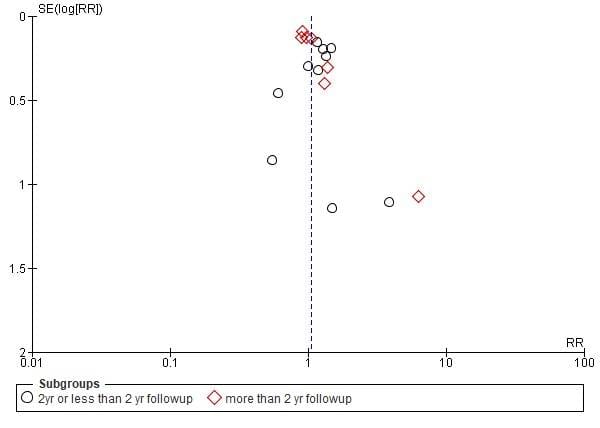


**S2.Cardiac death**


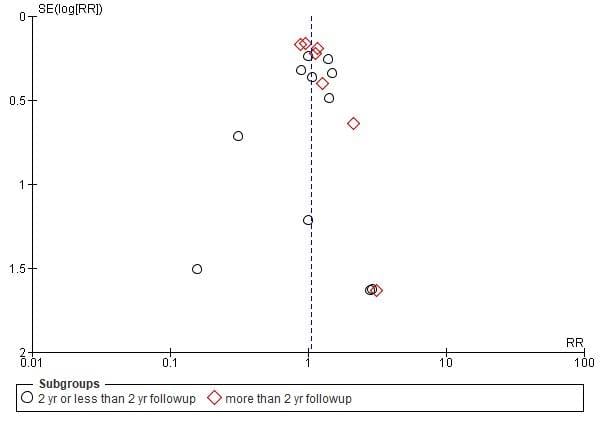


**3s. Target lesion revasculization**


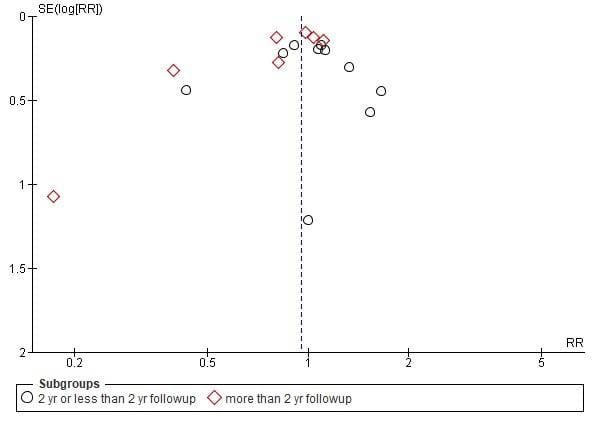


**4s. Late Stent Thrombosis**


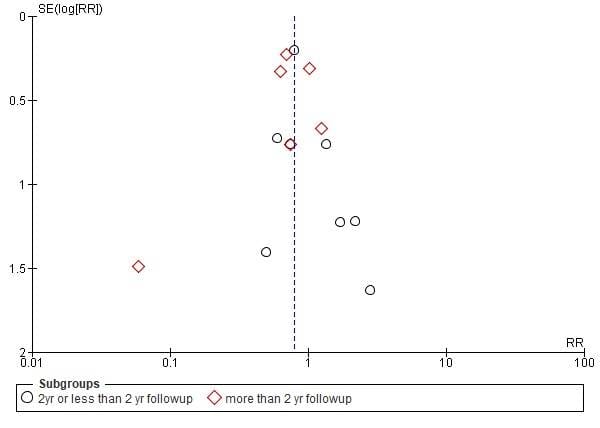


**5s. DOCE/TLF**


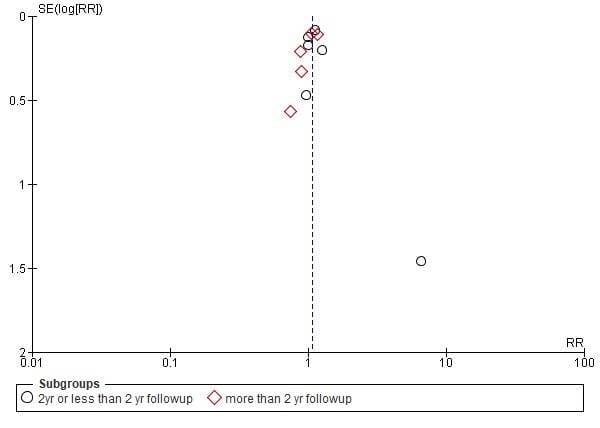


**6S. Risk of bias graph**


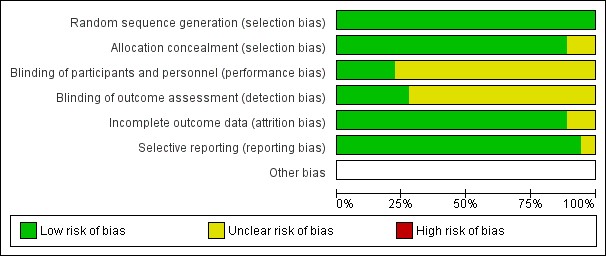


**7S.Risk of bias Summary**


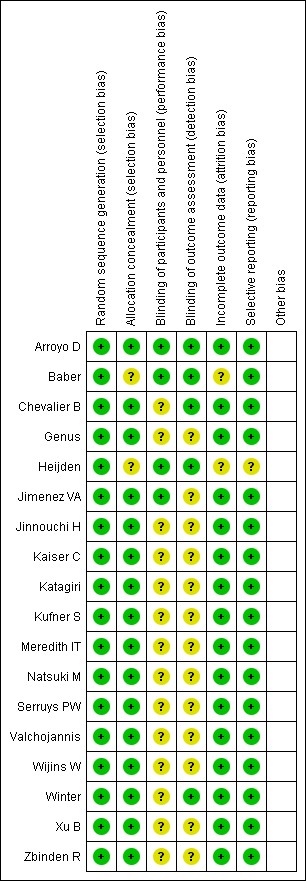

Supplement: Supplementary file 1 — Additional file 1. [file 43044_2024_522_MOESM1_ESM.docx]
